# Quantification of UV Light-Induced Spectral Response Degradation of CMOS-Based Photodetectors

**DOI:** 10.3390/s24051535

**Published:** 2024-02-27

**Authors:** Pablo F. Siles, Daniel Gäbler

**Affiliations:** X-FAB Global Services GmbH, 99097 Erfurt, Germany; daniel.gaebler@xfab.com

**Keywords:** UV sensing, photodiodes, UV degradation, spectral response, UVC, sterilization

## Abstract

High-energy radiation is known to potentially impact the optical performance of silicon-based sensors adversely. Nevertheless, a proper characterization and quantification of possible spectral response degradation effects due to UV stress is technically challenging. On one hand, typical illumination methods via UV lamps provide a poorly defined energy spectrum. On the other hand, a standardized measurement methodology is also missing. This work provides an approach where well-defined energy spectrum UV stress conditions are guaranteed via a customized optical set up, including a laser driven light source, a monochromator, and a non-solarizing optical fiber. The test methodology proposed here allows performing a controlled UV stress between 200 nm and 400 nm with well-defined energy conditions and offers a quantitative overview of the impact on the optical performance in CMOS-based photodiodes, along a wavelength range from 200 to 1100 nm and 1 nm step. This is of great importance for the characterization and development of new sensors with a high and stable UV spectral response, as well as for implementation of practical applications such as UV light sensing and UV-based sterilization.

## 1. Introduction

A wide number of the CMOS-based photodetectors in use today have no (or very poor) sensitivity in the UV spectral range, mainly because the backend layers are absorbing the UV light. The junction design is also an important factor, but more likely for degradation effects. Nevertheless, UV light sensing has become in recent years a topic of increasing interest, due to the surge of a plethora of new technological applications, such as sterilization, UV spectroscopy, biological analysis, space imaging, UV-based cure processes, and more [1,2,3,4,5,6,7,8]. Although, there are still a few major challenges in the field of UV light sensing. One of these challenges is regarding the typical low sensitivity to UV light due to the short penetration depth in Si. This means that most of the photo-generated carriers within the upper atomic layers in Si cannot be detected because of the strong recombination through the interface states. Several approaches dealing, for example, with the photodiode dopant profile [9,10] or the engineering of photodetectors optimizing design and manufacturing processes have made it possible to reach not only high UV spectral response [11,12] but also to address the poor stability or spectral response degradation due to exposure to UV light conditions. Achieving such optical robustness under high-energy UV-light illumination conditions is certainly the second major challenge in UV light sensing. Such degradation mechanisms may be explained due to trap generation [13] or changes in the fixed charges and the interface states at the Si/SiO_2_ interface above the photodetector, originating from the very high photon energy (6.2–4.1 eV for wavelengths between 200 and 300 nm). There exists also a plethora of approaches for the development of UV photodetectors, which include the usage of compound semiconductors with a wide bandgap such as SiC or ZnO [14,15], usage of Silicon-in-Insulator (SOI) structures with a shallow surface detection layer [8], the proper tunning of doping levels to reach high concentration surface layers near the Si surface [16], back side illumination structure approaches, or other thin-film or nanostructure-based approaches [17]. Our more recent research on the development of new UV photodiodes, using X-FAB’s Semiconductors Foundries XS018 technology, shows a significant spectral response improvement especially for wavelengths around 260 nm, which is relevant for applications in sterilization. It also shows a remarkable robustness (<5% degradation) under UV light stress conditions.

The UV instability of Si-based photodetectors is certainly a very complex phenomenon, which may depend on several parameters such as irradiance, duration of UV light exposure, radiant exposure, wavelength of the UV radiation, type of photodetector, etc. [18]. Therefore, the robustness of photodetectors must be investigated systematically, with a reliable measurement methodology which covers a wide range of experimental parameters. It is important to precisely quantify the spectral response degradation due to UV light exposure, at any stress wavelength of interest. In addition, it is also very important that the impact on the optical performance of photodetectors is determined not only at the wavelength of exposure but also along the entire spectral range where such photodetectors are expected to be used. It then becomes necessary to count with a reliable, fast, and standardized measurement methodology which allows us to perform such systematic characterizations. Due to the lack of a common standard, the present work intends to propose a systematic measurement methodology for the investigation and quantification of spectral response degradation due to UV light exposure in CMOS-based photodetectors. It is also to determine the overall impact on the optical performance of such photodetectors along the entire spectral range of operation, while maintaining always well-defined energy conditions for the UV exposure.

## 2. Materials and Methods

All the photodetectors characterized and presented in this work were fabricated with X-FAB’s Semiconductors Foundries XS018 technology [19]. Data are shown for photodiodes belonging to three different main modules: module A, which offers a good spectral sensitivity over a broad wavelength range; module B, which provides specially outstanding spectral response in the human eye response range, with its maximum point of sensitivity close to the green region; and module C, which is specially dedicated to performing with a high and stable spectral response in the UV range. Table 1 briefly summarizes the different XS018-based photodetectors for which experimental UV stress and spectral response degradation investigations are shown in this work.

**Figure 1:**
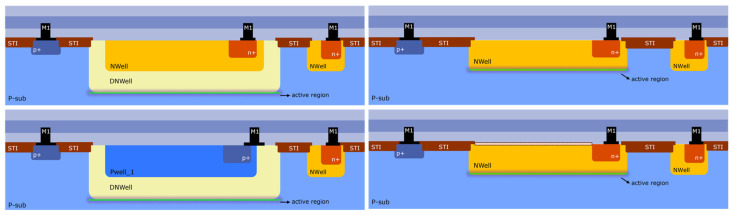
Conceptual cross-section diagram of the different CMOS-based photodiodes investigated in this work. In accordance with the naming shown in Table 1, top-left corresponds to doa, bottom-left corresponds to dob, top-right corresponds to doe, and bottom-right corresponds to doeher.

Figure 1 shows a conceptual diagram of most of the devices listed in Table 1. All photodetectors are fabricated following X-FAB’s XS018 process flow technology. All devices can be fabricated scalable in size, as well as in arrays of photodiodes. Nevertheless, for the investigations performed here, all measurements are performed on single devices. For simplification, all front-end layers which comprise different metallization levels (M2, M3, etc.) and the passivation layer are not shown in the diagrams. Some of these metallization levels may be used as a light shield, for example, to avoid photons reaching other regions instead of the photodiode’s pn-junction active region. All devices are built over a bulk p-type substrate, where the electrical connection is realized via a p+ implant and a metal contact at the M1 level. In the case of the doa device, the pn-junction proper depth is realized via an NWell plus a DNWell (deep NWell), which can be contacted via an n+ implant and a metal contact at the M1 level. The dob device conforms its pn-junction also via a DNWell. Although, in this case, the electrical connection is realized via a p+ implant, once the DNWell is pinched to a PWell_1. On the other hand, the doe and doeher devices realize the pn-junction only via an NWell, where electrical contact is possible thanks to an n+ implant and a metal contact at the M1 level. Different from the doe device, the doeher photodetector possesses a special layer at the back end with special optical properties which allows precise optimization of the optical performance, especially in the Visible and NIR spectral ranges. As described in Table 1, the UVC photodetector is realized by a special structure comprised by two pn-junctions, the PWell_2 to DNWell upper junction and the DNWell to p-Sub lower junction.

### 2.1. Test Setup

The photocurrent measurements were performed in situ, during illumination UV conditions, using a Keithley 4200A DC Parameter Analizer (Tektronix, Beaverton, OR, USA). All measurements have been performed at 27 °C (slightly elevated room temperature, to ensure precise temperature control). Wafers were placed on the chuck of a Summit 200-FA-AP probe station (Form Factor GmbH, Thiendorf, Germany). All photodetectors were operated with standard specification conditions, with V_Cathode_ = 0.9 V, V_Guard_ = 3.3 V and V_Anode_ = 0.0 V (substrate/bulk/chuck).

### 2.2. UV Stress Methodology

Conventional artificial UV light sources such as UV lamps were found to be inconvenient for UV degradation wafer-level investigations of photodetectors. On the one hand, the global illumination may affect other light-sensitive test structures and the close electrical contact via probe-wedge may induce undesired light reflections, when the illumination is performed in situ. On the other hand, such light sources provide a poorly defined energy spectrum, making it challenging to define the specific wavelength of illumination and consequently quantifying at which wavelength (and intensity) the UV stress is applied. Despite the points mentioned here, in the case of UV lamps, it is always possible to devise a way to normalize the illumination area and dose onto the photodetector under test. In fact, in most research investigations about UV degradation, often an intensity value per area is given, but without a complete spectrum. So, it remains unknown at which wavelength (and intensity) the UV stress is provided. Due to the typical broadband nature of a UV lamp as well as variations on the spectral output, a UV lamp does not provide stress at all wavelengths of the UV spectrum. Other light sources such as UV LEDs could also be used, but such LEDs provide non-uniform light, which brings other technical challenges to improve the illumination uniformity. Nevertheless, in this case, the illumination is also global, which may also induce undesired light reflections.

**Figure 2:**
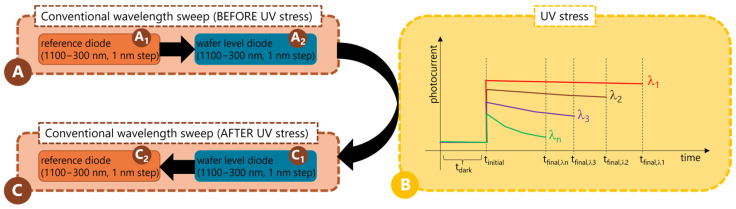
General scheme for photocurrent measurements before (**A**) and after (**C**) the UV stress methodology (**B**). Differently colored lines indicate different stress wavelengths.

To perform the optical characterization of the photodetectors and to quantify the impact on their optical performance due to UV stress, the spectral response is measured before and after a specially devised UV stress step. Here, a laser-driven light source (Model EQ-99X LDLS, Energetiq (Wilmington, MA, USA), a monochromator (Hyperchromator, Mountain Photonics GmbH, Landsberg am Lech, Germany), and a non-solarizing optical fiber (Multimode Solarization Resistant Optical Fiber, 0.22 NA, Ø105 µm core, Thorlabs, Newton, NJ, USA) are used in a customized manner. A reference detector is also used before or after every wavelength sweep, as indicated in Figure 2. This allows for a precise estimation of the light power intensity brought via the optical fiber onto the photodetector. In addition, a second control detector continuously monitors the output at the light source to counter for possible fluctuations or variations due to aging and other factors. For every conventional wavelength sweep, a dark current correction is always performed. The monochromator receives the white light output of the laser-driven light source and splits the light into every wavelength between 200 nm and 1100 nm (with a smaller step possible of 1 nm), thanks to a special optical set up which comprises the usage of proper filters, mirrors, and gratings. This allows bringing, via the non-solarizing fiber, any desired wavelength only onto the photodetector of interest, avoiding the illumination (or stress) of other devices as well as undesired light reflections. 

The spectral response measurements before (step A) and after (step C) the UV stress step (step B) correspond to standard monitoring of the photocurrent level as the wavelength is swept between 1100 nm and 300 nm, with 1 nm steps (2 nm FWHM). Such measurement before the UV stress (step A) includes a wavelength sweep on a reference calibrated diode (which allows the determination of the light power intensity) and a subsequent wavelength sweep on the photodetector of interest (wafer-level). 

For the UV stress step (step B), it is ensured that the UV stress methodology is realized under well-defined energy conditions. The wavelength is fixed at different values between 400 nm and 200 nm, with a step as small as 1 nm (2 nm FWHM). The stress time at each wavelength is defined in such a way that the exact illumination conditions (fluence rate) are maintained. It is important to mention that the UV stress is applied locally and is delimited only by the optical fiber diameter (~105 nm), which means that not the entire photodetector has been stressed during the illumination. Also, to perform a reliable UV stress and subsequent quantification of the photodetector’s degradation, the spectral response measurement after the UV stress (step C) must be performed in a reversed order with respect to the initial measurement (step A). This means that first, the wavelength sweep is performed on the photodiode of interest (wafer level), and subsequently, the wavelength sweep on the reference diode is performed. In this way, it is ensured that the electrical contact always remains the same during the entire process and that the optical fiber also does not change its position over the photodetector. These in situ pre/post optical photocurrent measurements, as well as the UV stress step, are summarized in detail in Figure 2.

### 2.3. UV Stress Methodology Applied on CMOS-Based Photodetectors

**Figure 3:**
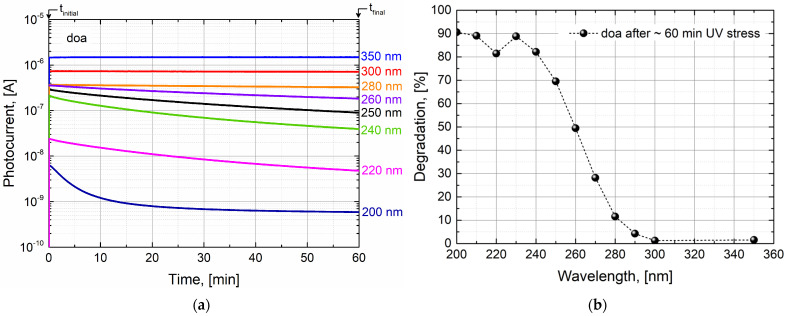
UV stress methodology for CMOS photodetectors. Data are shown for the case of module A, doa photodiode: (**a**) over-time photocurrent monitoring under UV stress, shown for some pre-defined wavelength values; (**b**) estimation of the absolute spectral response degradation suffered at each stressing wavelength. Dotted line serves merely as a guide-to-the-eye to follow the tendency of the UV degradation behavior.

As mentioned previously, the UV stress is carried out at pre-defined fixed stressing wavelengths, between 400 nm and 200 nm (see Figure 3 for some wavelength examples between 200 nm and 350 nm). At such wavelength values, the photocurrent is monitored over a specific stress time (t_stress_ = t_final −_ t_initial_). A dark current correction is also performed by subtracting the average dark current which is measured for about 10 s (t_dark_) before the shutter is opened (without light exposure). The degradation of the spectral response (shown in % in Figure 3b) is calculated from the variation in photocurrent measured at the final measurement time (t_final_) and the initial measurement time (t_initial_). For all the photodiodes tested in this work, it is observed that shorter wavelengths (therefore more photon energetic) have a higher degradation potential. In the device case shown in Figure 3, such degradation reaches some level of saturation at around 90% close to 200 nm. Also, for all the photodiodes tested in this work, for wavelengths > 300 nm, there is no evident spectral response degradation noticed due to UV light stress.

**Figure 4:**
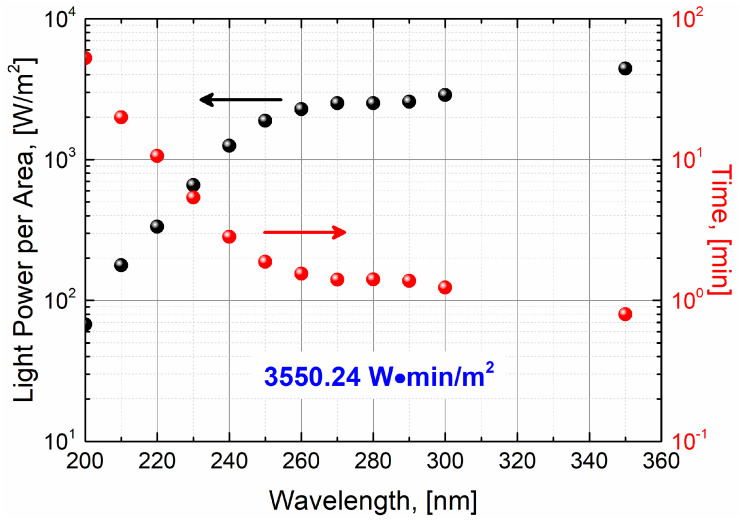
Light power per area dependency on wavelength and determination of UV stress exposure times for a constant fluence rate of 3550.24 W·min/m^2^. The colored arrows indicate the corresponding proper axis of light power per area (black) and time (red).

The light power per area reaching the photodetector varies with the stress wavelength used, as shown in Figure 4. Also, as shown in Figure 3, different stress levels (and therefore UV degradation) can be induced at different stress wavelengths. Therefore, to ensure a fair comparison and evaluation in terms of UV degradation of different photodetectors, as well as the stress induced at every stressing wavelength, it is necessary to properly control the dosage of UV light reaching the photodetector at every stress wavelength. The approach in this work is to expose the photodetectors to a wide range of stress wavelengths while always maintaining the same light illumination conditions. In this way, no UV wavelength is favored, and each stress wavelength will induce the same stress over the photodetector. 

Therefore, we employ a constant light fluence over the light-exposed surface of the photodetector. This is known as the fluence rate or radiant exposure and is defined as the product of the power of the UV electromagnetic radiation incident on a surface per unit surface area and the duration of the light exposure or simply the product of the effective light irradiance and the UV stress time [20]. For simplicity, and in accordance with some experimental measurement requirements, such as reasonable UV stress and measurement times, in this work, we defined a fluence rate of 3550.24 W·min/m^2^. Consequently, the exposure times at every stress wavelength must be adjusted accordingly. Table 2 summarizes the stress wavelengths used during the UV stress procedure (step B in Figure 2), as well as their corresponding exposure times. This is also displayed in Figure 4.

## 3. Results and Discussions

### 3.1. Quantification of Optical Degradation Due to UV Light Exposure

**Figure 5:**
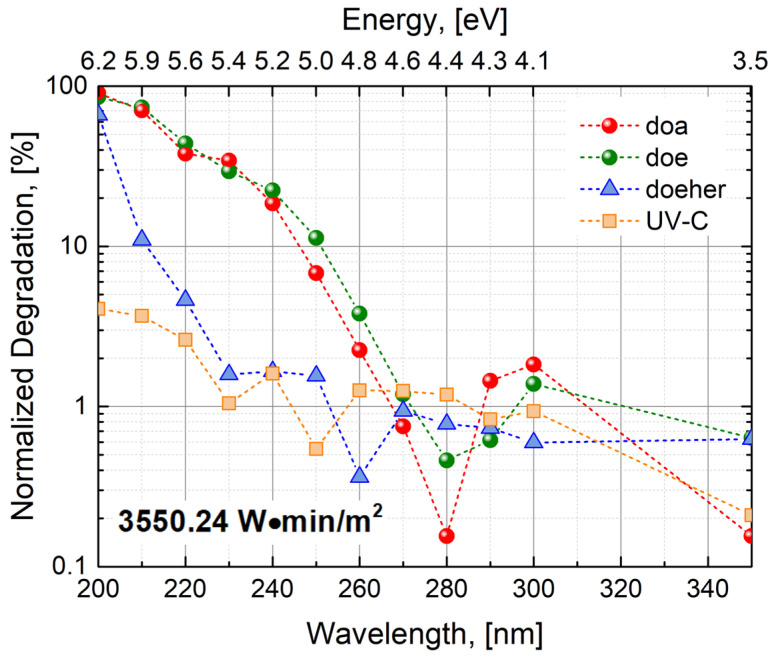
Quantification of the degradation induced due to UV light exposure, considering stress wavelength between 400 nm and 200 nm. Data are shown for some representative examples including photodetectors with a wide range of spectral applications.

Once an adequate fluence rate is defined, it is then required to re-normalize the estimations of UV degradation shown in Figure 3 with precise UV exposure times to guarantee the same UV light exposure conditions at each stress wavelength. The results of such normalization are shown in Figure 5, considering the case of the doa device (previously shown in Figure 3), as well as other examples of X-FAB’s photodetectors (doe, doeher, and UVC) designed for operation in a variety of spectral ranges (see Table 1 for further details). In general, these results confirm that non-UV photodetectors should not be exposed to stress wavelengths below 300 nm. Degradation effects become more evident around 260 nm, growing exponentially as the photo energy increases and reaching almost a total loss of optical sensitivity at 200 nm. Due to some structural characteristics, some non-UV photodetectors (see doeher type in Figure 5) appear to be slightly more resistant to UV light exposure. Nevertheless, these devices also suffer almost a total loss of optical sensitivity as the stress wavelength approaches 200 nm. A remarkable highlight is the robustness of the UV photodetectors under UV stress conditions, where a spectral response degradation below 5% is observed for the more energetic wavelength applied in this work. Due to technical limitations, and to ensure good reliability of the experimental results presented here, 200 nm is the lower limit investigated in this work. For wavelengths below 200 nm, not only the stability of the light source system may be compromised, but also strong solarization effects are observed in the optical fiber. This is caused by the formation of absorbing centers due to the intense UV light flux. Therefore, it is no longer possible to properly bring the light into the photodetectors without avoiding undesired significant light loss.

### 3.2. Overall Impact of Optical Performance for an Extended Spectral Range

Once a robust methodology has been laid down to quantify the degradation of sensitivity suffered by CMOS-based photodetectors due to UV stress, it is of great interest to determine the impact that such degradation has on the overall optical performance of such devices. Particularly, when such an impact may possibly be irreversible (to the best of the author’s knowledge, there is no clear evidence that a UV degradation mechanism can be reversed). The undesired or unproper exposure to UV light may adversely compromise the safe and successful outcome of the specific technological application in which a silicon-based photodetector may be implemented. Under UV light exposure, the optical performance of a photodetector is impacted, not only in the UV wavelength range but certainly in the entire spectral range. This would turn the device unresponsive, even in specific spectral ranges where such a device is expected to offer its maximum light sensing capabilities.

**Figure 6:**
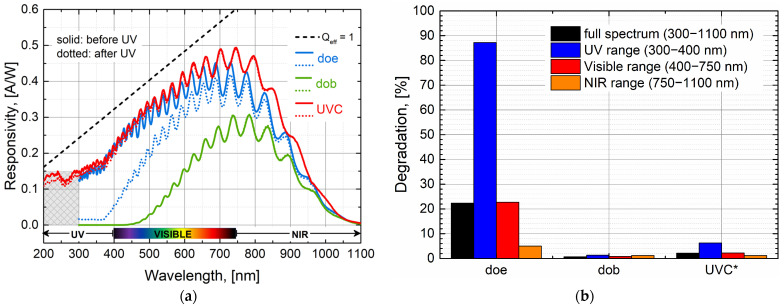
Overall impact on the optical performance of CMOS-based photodetectors due to UV stress light exposure: (**a**) Photodetectors responsivity before (solid data) and after (dotted data) UV stress conditions. Dashed lines correspond to the ideal responsivity (Q_eff_ = 1). (**b**) Estimation of the spectral response degradation suffered along different spectral ranges of interest. (*) For the case of the UVC photodetector the labels for spectral ranges should be read as full spectrum (200–1100 nm) and UV range (200–400 nm), once the experimental data collection starts at 200 nm instead of 300 nm.

Figure 6 shows the example of three photodetectors which were submitted to the UV stress methodology proposed in Section 2.3 and following the test sequence shown in Figure 2. Solid data represent conventional wavelength sweep measurements before UV stress (step A, Figure 2); meanwhile, dotted data represent conventional wavelength sweep measurements after UV stress (step C, Figure 2). Dashed back data indicate the ideal spectral response of a photodetector, which would be the ideal case when its quantum efficiency is equal to one. Each photodetector in Figure 6 was selected to showcase three main particular cases. The first case corresponds to a non-UV photodiode (doe), which is severely affected under UV stress illumination conditions (especially for wavelengths below 260 nm), as shown also in Figure 5. After UV stress, the optical performance of the device is severely impacted along the entire spectral range measured (1100 nm to 300 nm). The strongest impact is clearly in the UV range, where a degradation of about 88% is observed. Note that the UV range for this device corresponds to wavelengths only from 400 nm to 300 nm, once such non-UV photodiodes are not specified to be operated below 300 nm. Nevertheless, the responsivity of the device is also affected beyond the UV range, about 23% in the Visible range and about 5% in the NIR range. The second case corresponds also to a non-UV photodiode, which is especially designed to be responsive for the Red and NIR spectral range (dob). This device is designed to be non-responsive in the UV spectral range. Therefore, no impact on its optical performance is expected to appear due to UV stress light illumination, as clearly shown in Figure 6. The third case considered here is a newly developed UV photodetector (UVC), which shows high UV response down to 200 nm. This is the lower wavelength operation limit specified for these kinds of photodetectors. Such devices also show a strong robustness under UV stress conditions, with a maximum responsivity degradation of 5–6% in the UV spectral range. Such degradation is almost unnoticeable in the Visible and NIR spectral ranges (below 3% in both cases). For a fair comparison, it should be noted that the result shown in Figure 6b does not imply that a photodetector like the dob is more robust to UV light conditions compared to the UVC photodetector. The low degradation for dob (in %) is because such a device is specially designed to be unresponsive for wavelengths below ~450 nm. Therefore, the photocurrent levels and variations before and after UV stress illumination conditions are extremely small in this case.

**Figure 7:**
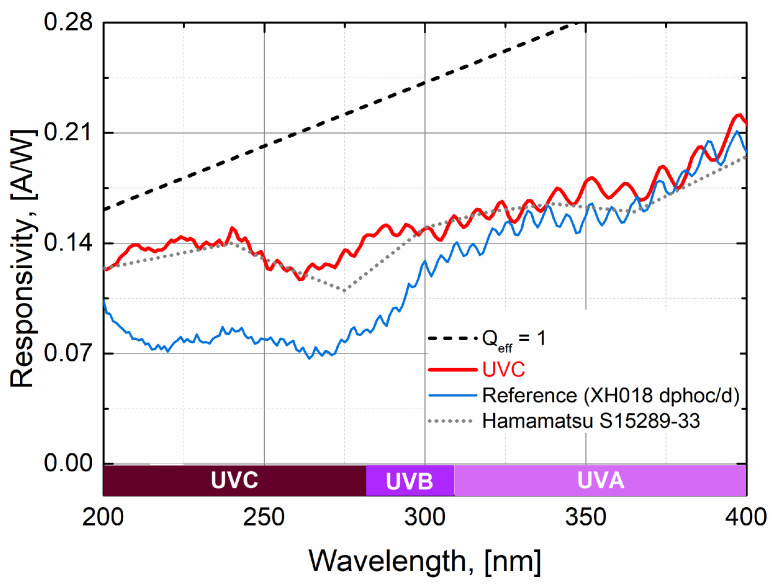
Optical performance in the UV spectral range for the XS018 technology-based UVC photodetector, in comparison to other X-FAB’s technologies (XH018) as well as other discrete back side illuminated commercially available photodetectors.

As a further comparison of the improved optical performance shown by the UVC photodetector, a comparison is made with other previous X-FAB’s technologies such as the XH018 technology-based UV detector. Figure 7 shows the clear improvement in optical performance in the UV range for the UVC detector (red solid data), in comparison to the XH018 UV detector (blue solid data). Such improvement starts to be more evident below 300 nm, and especially of interest is the performance around 260 nm, which opens up important possibilities for applications of UV sterilization. As a comparison, the case of a commercially available discrete back side illuminated UV photodetector (gray dotted data) is also shown. Nevertheless, it must be pointed out that such discrete devices are based on a back side illumination technology, which is very different from the technology approach for the case of the UVC photodetector, based on X-FAB’s CMOS XS018 technology. Also, the data for this case (at 25 °C) are obtained from available public documentation and such a device has not been directly measured with the measurement methodology approach proposed in this work. Therefore, this serves solely as a first general performance comparison.

## 4. Conclusions

As expected, different photodetectors are impacted differently under UV stress illumination conditions, depending on the specific technical application for which they are designed. This also defines aspects such as photodiode structure, design or layout characteristics and even adequate operation conditions. Therefore, it becomes very important to develop a robust measurement methodology which allows a reliable characterization of CMOS-based photodetectors in terms of UV stress and degradation. The work presented here is the result of several optimization loops in terms of electrical and optical measurements methodologies which are easily applied to any kind of front-side illuminated photodetector in a fully automated manner. In the case of the UV photodetectors shown here, designs and process flavors have been optimized to achieve high spectral responsivity and robustness under UV light illumination conditions. Compared with X-FAB’s technologies (XH018) and even other discrete backside illuminated devices, we have observed a significant spectral response improvement, especially for wavelengths around 260 nm, which is of great interest for UV-based sterilization applications. Our planned research on UV light sensing applications continues and will also include the influence of operation temperature on spectral response degradation performance due to UV stress conditions, as well as the impact on other important photodetector parameters such as dark current (leakage current) and capacitance.

## 5. Patents

Four patents related to UV light sensing are pending to protect the IP provided by X-FAB regarding the development and realization of silicon-based photodetectors with an exceptionally high UV spectral response.

## Figures and Tables

**Table 1:** X-FAB’s XS018 technology-based photodetectors investigated in this work.

Photodetector	Basic Structure	Sensitivity Application
doa	Module A, DNWell/p-Sub	Wide spectral range
dob	Module A, DNWell (pinched to PWell_1)/p-Sub	Red and NIR spectral range
doe	Module A, NWell/p-Sub	Wide spectral range
doeher	Module B, NWell/p-Sub	Enhanced Visible and NIR
UVC ^1^	Module C, PWell_2/DNWell/p-Sub	Enhanced for UV range

^1^ This new photodetector is soon to be released as dosuv.

**Table 2:** UV light exposure stress times applied at each stress wavelength.

Stress Wavelength (nm)	Exposure Time (s) ^1^
200	3151.8
210	1197.0
220	637.3
230	322.9
240	170.0
250	112.8
260	93.2
270	84.6
280	84.8
290	82.8
300	74.1
350	48.0

^1^ This is precisely controlled by an automated optical shutter mechanism.

## Data Availability

Raw data related to the measurements of the photodetectors shown here are available. Specimens of all different fabricated photodetectors as well as demonstration kits can be provided to interested parties under individual agreements.
